# Louisville Odontological Society

**Published:** 1896-05

**Authors:** C. R. Shacklitle

**Affiliations:** Secretary; Louisville, Ky.


### Domestic Correspondence.

### LOUISVILLE ODONTOLOGICAL SOCIETY.

Louisville, Ky., April 2, 1896.

To the Editor:

Dear Sir,—At a gathering of the younger members of the dental profession of this city, during the latter part of 1895, it was, after some discussion, considered advisable to form a local society for the encouragement of social and professional advancement. As a result "The Louisville Odontological Society" was inaugurated, and meets the first Saturday of each month.

The interest and benefit from these meetings has been marked from the start. The next meeting will be with Dr. J. H. Harrington, at the Kenton Club, the first Saturday in April.

The following officers were elected: President, Dr. W. E. Grant; Vice-President, Dr. M. M. Eble; Secretary-Treasurer, Dr. C. R. Shacklitle.

C. R. Shacklitle,
*Secretary.*

